# Relationship between Anticholinergic Burden and Health-Related Quality of Life among Residents in Long-Term Care

**DOI:** 10.1007/s12603-020-1493-2

**Published:** 2020-10-23

**Authors:** Ulla L. Aalto, H. Finne-Soveri, H. Kautiainen, H. Öhman, H.-M. Roitto, K.H. Pitkälä

**Affiliations:** 1 Dept of Social Services and Health Care, Helsinki Hospital, Home-care Services, PO BOX 6540, FI-00099, City of Helsinki, Finland; 2 Department of General Practice, University of Helsinki, Helsinki, Finland; 3 National Institute of Health and Welfare, Helsinki, Finland; 4 Unit of Primary Health Care, Helsinki University Hospital, Helsinki, Finland

**Keywords:** Anticholinergic burden, long-term care, nursing home, HRQoL, dementia

## Abstract

**Objectives:**

Anticholinergic burden defined by the Anticholinergic Risk Scale (ARS) has been associated with cognitive and functional decline. Associations with health-related quality of life (HRQoL) have been scarcely studied. The aim of this study was to examine the association between anticholinergic burden and HRQoL among older people living in long-term care. Further, we investigated whether there is an interaction between ARS score and HRQoL in certain underlying conditions.

**Design and participants:**

Cross-sectional study in 2017. Participants were older people residing in long-term care facilities (N=2474) in Helsinki.

**Measurements:**

Data on anticholinergic burden was assessed by ARS score, nutritional status by Mini Nutritional Assessment, and HRQoL by the 15D instrument.

**Results:**

Of the participants, 54% regularly used ARS-defined drugs, and 22% had ARS scores ≥2. Higher ARS scores were associated with better cognition, functioning, nutritional status and higher HRQoL. When viewing participants separately according to a diagnosis of dementia, nutritional status or level of dependency, HRQoL was lower among those having dementia, worse nutritional status, or being dependent on another person's help (adjusted for age, sex, comorbidities). Significant differences within the groups according to ARS score were no longer observed. However, interactions between ARS score and dementia and dependency emerged.

**Conclusion:**

In primary analysis there was an association between ARS score and HRQoL. However, this relationship disappeared after stratification by dementia, nutritional status and dependency. The reasons behind the interaction concerning dementia or dependency remain unclear and warrant further studies.

## Introduction

Older people residing in long-term care are living the last years of their lives (1). They are likely to have cognitive impairment, deficits in physical functioning, and multiple coexisting diseases (1, 2). Their diseases are often chronic (2), and thus, the aim of the care is in most cases palliative, i.e. to maintain or improve quality of life (QoL). Health-related quality of life (HRQoL), recognized as a relevant aspect of QoL, consists of physical, emotional, and social dimensions associated with a person's illnesses and their treatment (3).

Potentially inappropriate medications are widely used among older people living in long-term care facilities (4, 5). Most drugs with anticholinergic properties (DAPs) are considered potentially inappropriate according to American Geriatrics Society Beers criteria (6), and thus, should be avoided in older adults. Despite this recommendation, DAPs remain frequently used among older nursing home residents (7, 8, 9, 10, 11). DAPs may cause adverse effects on both central and peripheral nervous systems (12). Dry mouth, dry eyes, constipation, dizziness, cognitive decline, and falls are some of the common adverse effects (13, 14). Several studies have reported an association between anticholinergic burden and physical impairment (15). In a recently published study, even a relationship between use of certain DAPs and increased risk of dementia has been suggested (16). Polypharmacy among older people living in long-term care is common (17), leading easily to a situation in which several DAPs are present, increasing the anticholinergic burden. Unwanted side-effects tend to increase hand in hand with greater anticholinergic burden (18).

There is a scarcity of studies exploring the relationship between anticholinergic drug use and HRQoL in older people, and the results are somewhat inconclusive. Studies among community dwelling older people have reported an association between anticholinergic drug use and the lower physical component score of HRQoL according to SF-12/SF-36 (19, 20). In studies among older long-term care residents with high prevalence of cognitive impairment, exposure to anticholinergic and sedative drugs defined by the Drug Burden Index has shown to have an inverse association with QoL (5, 21). However, in one American study investigating a nursing home population, no association between anticholinergic burden and engagement in activities, an important indicator of QoL, was observed (7). There is some evidence, that among older long-term care residents DAP use is associated with decreased well-being, which can be considered an aspect of HRQoL (22, 23).

Despite the importance of the topic, limited research exists on the association between DAP use and HRQoL among long-term care residents. Our hypothesis was that greater anticholinergic burden would have an association with lower HRQoL. The rationale behind our hypothesis was that the common side-effects of DAPs may be associated with lower HRQoL. Especially residents with dementia are likely to be vulnerable to the harmful effects of DAPs such as cognitive decline (24).

The aim of our study was to investigate the association between anticholinergic burden, defined by the Anticholinergic Risk Scale (ARS) (14), and HRQoL according to 15D (3). A second aim was to determine whether certain underlying conditions such as dementia, nutritional status or dependency on personal care have an interaction with the relationship between 15D and ARS score.

## Methods

All residents living in all long-term care facilities (including nursing homes and assisted living facilities) in Helsinki in 2017 were invited to participate (N=3895). Exclusion criteria were patient refusal, or having dementia and no proxy available to give informed consent. Because the aim was to assess the burden arising from DAP use, we excluded residents not using regularly any medications or those whose medication list was not available. After exclusions, 2474 participants remained.

A trained, registered nurse performed the assessments and interviews according to a structured study protocol. The data were collected in March 2017, and each resident was assessed over the course of one day. The medication list was a point-prevalence on the same day. Medications were retrieved from participants' medical charts.

All medications used were classified by the Anatomical Therapeutic Chemical (ATC) Classification System (25). Only medications administered regularly were included. As described in previous research, regularly used drugs were considered to be those for which there was a documented regular sequence of administration (26). Thus, medications given pro re nata, were not taken into account. DAPs were defined by the ARS, which is a list of 49 commonly used drugs with anticholinergic potential (14). The ARS ranks medications on a 3-point scale (0=limited or none; 1=moderate; 2=strong; 3=very strong anticholinergic potential). The total ARS score is the sum of the points of individual medications. However, the scale does not consider the dosage of an individual drug. Among older people, even a moderate change in the ARS score has been suggested to have an association with cognitive and functional decline (27); hence, we grouped the participants into four groups from none (G0) to very strong (G3) anticholinergic potential defined by the ARS to view also the participants with moderate anticholinergic burden. The ARS has been used in several studies particularly involving nursing home residents, and it has been shown to have an association with outcomes in cognitive performance and physical function (9, 28, 29).

In each long-term care unit, background data on demographics, medical diagnoses (acute illnesses and chronic conditions) were retrieved from medical charts.

The severity of dementia was assessed by using the Clinical Dementia Rating (CDR) “memory” item [30] and the Mini Mental State Examination (MMSE) (31). The CDR “memory” item grades stages of cognition on a 5-point scale (0=no memory loss; 0.5=consistent slight forgetfulness; 1=moderate memory loss; 2=severe memory loss; 3=severe memory loss, only fragments remain) (30). MMSE is a screening tool for dementia, which also can be used in measuring cognitive impairment, yielding a score from 0 to 30 (31). Mini Nutritional Assessment (MNA) was applied to assess participants' nutritional status (32). Dependency in activities of daily living (ADL) was assessed by a 4-point scale according to the CDR “personal care” item (1=totally independent; 2=needs prompting; 3=requires assistance in dressing, personal hygiene, and keeping of personal belongings; 4=requires much help with personal care, often incontinent) (30). Those in groups 3 and 4 were considered to be dependent for personal care.

The main outcome of our study was HRQoL assessed by the 15D instrument, which explores health state in 15 dimensions (mobility, vision, hearing, breathing, sleeping, eating, speech, excretion, usual activities, mental function, discomfort and symptoms, depression, distress, vitality, sexual activity) (3). The 15D is a validated instrument for use in various populations and health problems (3). It is primarily meant to be filled in by the respondent, but interview or proxy administration is also possible. 15D can be generated to a 15D score as an index measure in which the minimum score is 0 (being dead) and the maximum score is 1 (no problems on any dimension) (3).

### Statistics

The results are presented as means with standard deviations (SDs) or as counts with percentages. To evaluate the relationship of anticholinergic burden with HRQoL, we divided the participants into groups according to anticholinergic burden defined by ARS score (14) as follows: GO, ARS score=0; G1, ARS score=1; G2, ARS score=2; and G3, ARS score ≥3. Furthermore, we stratified the respective groups according to dementia diagnosis, nutritional status and dependency for personal care to investigate whether they have an interaction with the relationship between ARS score and HRQoL.

Statistical significance for the unadjusted hypothesis of linearity across categories (quartiles) of ARS score and characteristics of the study participants were evaluated using the Cochran-Armitage test for trend, analysis of variance (ANOVA), and logistic (ordinal) models with an appropriate contrast. Adjusted (age, sex and CCI) relationship between ARS score and dementia diagnosis, nutritional status, and dependency in personal care with 15D was analyzed using two-way analysis of variance. In cases of violation of assumptions (e.g. non-normality), a bootstrap-type test was used. Normality of variables was evaluated graphically and using the Shapiro-Wilk W test. Stata 16.1 (StataCorp LP, College Station, TX, USA) was used for the analysis.

## Results

The participants were divided into four groups according to the ARS score [G0 (n=1149); G1, (n=768); G2 (n=347); G3 (n=210)]. Of all participants, 1149 (46%) did not use any ARS-defined DAPs, and thus had an ARS score of 0 (G0); 31% scored 1 point (G1), 14% 2 points (G2), and 8% 3 or more points (G3). A greater ARS score was associated with younger age and a lower proportion of females. Those having higher ARS scores suffered more often from depression, other psychiatric illnesses, and Parkinson's disease. The greater the ARS score, the greater the number of drugs used on a regular basis. The number of ARS-defined drugs increased alongside greater anticholinergic burden; in G1, the mean number was 1.0, in G2 1.6, and in G3 2.1. The most frequently used ARS-defined drug groups were antipsychotics (33%) and antidepressants (23%).

Altogether, 1899 residents (77%) had a dementia diagnosis, whereas 575 (23%) did not. Along with greater ARS scores, a decreasing trend in the proportion of participants with a diagnosis of dementia was observed: in G0 78%, in Gl 82%, in G2 75%, and in G3 56% (p<0.001). An increasing trend in MMSE points with greater ARS scores was observed, indicating better cognition, and CDR also suggested milder cognitive impairment with higher ARS scores. Residents with higher ARS scores had better nutritional status than those with a lower ARS score. A decreasing trend in dependency on personal care with a greater ARS score was observed. 15D scores showed an increasing trend with ARS scores, thus indicating better HRQoL among those with a greater anticholinergic burden (Table 1).

**Table 1:** Participant characteristics according to anticholinergic burden defined by the Anticholinergic Risk Scale (ARS)

	**ARS-score=0 (GO) N=1149 (46)**	**ARS-score=1 (G1) N=768 (31)**	**ARS-score=2 (G2) N=347 (14)**	**ARS-score≥3 (G3) N=210 (8)**	**P-linearity**
Females, n (%)	849(74)	574(75)	242(70)	142(68)	0.032
Age, years, mean (SD^a^)	83(8)	83(9)	82(9)	79(11)	<0.001
Education <8 years, n (%)	443(39)	273(36)	138(40)	80(38)	0.97
Dementia, n (%)	896(78)	626(82)	260(75)	117(56)	<0.001
Prior stroke, n (%)	296(26)	177(23)	63(18)	58(28)	0.21
Depression, n (%)	100(9)	99(13)	58(17)	41(20)	<0.001
Other psychiatric diagnosis, n (%)	44(4)	68(9)	77(22)	65(31)	<0.001
Parkinson disease, n (%)	37(3)	44(6)	36(10)	18(9)	<0.001
Arthrosis and arthritis, n (%)	276(24)	176(23)	79(23)	39(19)	0.12
Diabetes mellitus, n (%)	233(20)	136(18)	68(20)	53(25)	0.38
Coronary heart disease, n (%)	238(21)	149(19)	62(18)	24(11)	0.004
MMSE^b^, mean (SD^a^)	12.7(7.4)	13.2(6.9)	13.6(6.9)	16.3(6.6)	<0.001
MNA^C^, n (%)					<0.001
well-nourished (24-30 points)	156(15)	114(17)	71(22)	45(23)	
at risk for malnutrition (17-23.5 points)	667(64)	461(67)	209(66)	126(65)	
malnourished (<17 points)	221(21)	109(16)	36(11)	22(11)	
Dependent for personal care (CDR^d^ personal care class), n (%)	1017(91)	680(90)	297(87)	181(87)	0.015
Cognition (CDR^d^ “memory” item), n (%)					<0.001
0-1	425(37)	297(39)	157(45)	123(59)	
2	329(30)	280(37)	115(34)	46(23)	
3	352(32)	173(23)	68(20)	32(16)	
Number of drugs used regularly, mean (SD^a^)	7.8(3.5)	9.3(3.4)	9.8(3.3)	10.8(3.8)	<0.001
15D score, mean (SD^a^)	0.596 (0.132)	0.619 (0.128)	0.626 (0.132)	0.629 (0.116)	<0.001


a. SD, standard deviation; b. MMSE, Mini Mental State Examination (31); c. MNA, Mini Nutritional Assessment (32); d. CDR, Clinical Dementia Rating (30)

**Figure 1:**
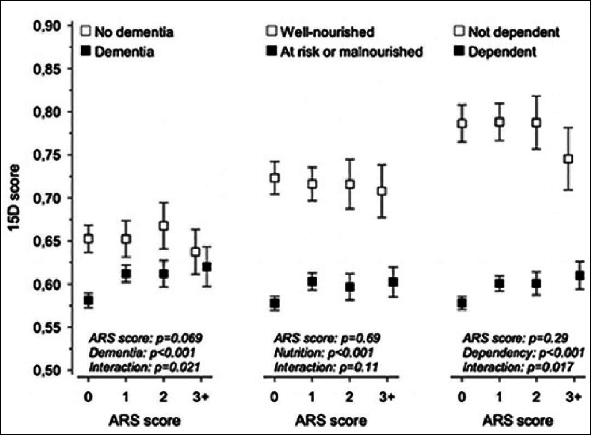
Relationship between anticholinergic burden measured by the Anticholinergic Risk Scale (ARS) score and health-related quality of life (HRQoL) measured by 15D score among residents with and without dementia diagnosis; at risk for malnutrition or malnourished and well-nourished; and dependent and not dependent. Adjusted for age, sex and Charlson Comorbidity Index (CCI)

To further investigate whether cognitive or functional decline had an interaction with the relationship between ARS score and HRQoL, we then stratified the participants according to a diagnosis of dementia, nutritional status, and those dependent on personal care. Between the ARS-score groups, significant differences in HRQoL were no longer observed. Overall, residents with dementia had lower 15D scores, indicating poorer HRQoL, versus those without dementia. Residents at risk of malnutrition or being malnourished had lower 15D scores than residents with good nutritional status (p<0.001), and the same pattern was observed in residents with functional dependency compared with those not dependent on personal care (p<0.001). Interactions between ARS score and dementia, and ARS score and dependency emerged, indicating different directions of trend in HRQoL in these two groups. Among those with dementia or dependency, 15D scores increased along with ARS scores, whereas they decreased among those without dementia and those who were not dependent. No significant interaction as regards nutritional status was observed (Figure 1).

We further explored the group of people with dementia. Among those with no anticholinergic burden (ARS score 0), the mean MMSE score was 11.2, whereas among those with dementia and ARS score ≥3, the respective figure was 13.7. Of those with dementia, most of the burden was associated with psychotropics, since 115/117 participants were on these drugs. Antipsychotic drugs were particularly common among them (80/117). Those without dementia suffered from psychiatric illnesses (n=46), stroke (n=24), Parkinson's disease (n=8), or ALS/MS (n=7). Nearly all of them were administered psychotropics (92/93) as well.

## Discussion

Our primary analysis suggested an association between higher anticholinergic burden and better health status in terms of cognition, nutrition, functioning and HRQoL. However, after stratification, no significant differences in HRQoL between the ARS-score groups remained. Furthermore, surprising interactions in the trends of 15D scores were observed in the groups stratified according to a diagnosis of dementia, and dependency as regards personal care. Among residents with dementia or dependency, the trend in HRQoL scores increased with greater ARS score, whereas the trend was in the opposite direction among residents without dementia and those who were not dependent.

The proportion of DAP users was consistent with those in earlier studies in which it has varied between 48% and 65% among nursing home residents (9, 33). The ARS score was associated with a higher number of drugs used, which is also in line with observations in previous studies (9, 34). We included only residents using at least one drug regularly, so the proportion of DAP users might be a slight overestimation of the true situation.

In the primary analysis, our finding of an increasing trend of HRQoL along with increasing anticholinergic burden was surprising and not in line with the results of previous studies (5, 21, 22, 23). Hence, we further stratified our sample according to various health conditions, ARS scores and HRQoL. After stratification and adjustments, the relationship between ARS score and HRQoL no longer existed. Irrespective of the ARS score, dementia, malnutrition and dependency were all associated with lower HRQoL, suggesting that these conditions are explanatory factors. These findings are in line with those in a prior study showing that the severity of dementia is associated with poorer QoL (35). Our findings concerning the associations between poorer HRQoL and malnutrition or dependency are also supported by existing evidence from previous studies (36, 37, 38).

The trend in ARS scores differed among those with and without dementia. Although it is challenging to determine whether it is the ARS score or the characteristics of participants that influences HRQoL, we explored the characteristics of our groups of participants. Those without dementia suffered from other psychiatric illnesses such as depression or chronic psychosis. Furthermore, almost all of them were administered psychotropic drugs. Thus, either the high burden of psychotropics is harmful in this patient group or the severity of these diseases has a negative impact on HRQoL. Because of the cross-sectional nature of the study it is impossible to draw definite conclusions on which explanation holds true.

On the other hand, among those with dementia and dependency the 15D score increased along with anticholinergic burden. It seems that participants with dementia and higher ARS scores had better cognition and functioning, thus resulting in higher 15D scores. Thus, those with the most severe dementia might be spared a high anticholinergic burden. A large proportion of those with high ARS scores had been given psychotropics and antipsychotics, suggesting that they might suffer from neuropsychiatric symptoms.

Our study has several strengths. To the best of our knowledge, it is the first one carried out in order to investigate the associations and interaction between anticholinergic burden, HRQoL and overall health status in connection with dementia, nutritional status and functional dependency among residents living in long-term care facilities. Our sample is large and well representative of older residents in long-term care in Helsinki. Because the medications were administered by nurses, drug use according to drug lists can be considered reliable. Data were collected from residents' medical records, and measurements were performed by trained nurses according to a standardized study protocol. The 15D instrument is a standardized and validated measure (3) showing high-level correlation with other HRQoL instruments such as EQ-5D and SF6D (39).

As a limitation, the cross-sectional nature of our study allows us only to report associations within the cohort. Although interactions and associations exist between ARS score, HRQoL and cognitive decline, nutritional status and dependency, it is not possible to draw any conclusions about causal relationships. Thus, we must emphasize that confounding by indication may be behind the findings of our study. Cognitive impairment in our population was common, and thus the 15D questionnaire was mainly completed by an interviewer or via proxy administration. Although caution in using a proxy respondent in measuring HRQoL has been suggested (40), the 15D instrument has been evaluated and shown to be reliable when used in this manner (3). Proxy administration enabled us to include persons with severe dementia, in order to have a more real-life spectrum of nursing home residents. Another limitation is that we used only one anticholinergic scale. While a number of scales exist, there is considerable discordance between them. Thus, the results and outcomes might have been different had a scale other than the ARS been used (28, 29). However, the ARS scale is widely used, particularly among nursing home residents (29).

## Conclusion

Whereas in the primary analysis there was an association between anticholinergic burden defined by the ARS, and HRQoL measured by the 15D instrument, this relationship disappeared when the participants were stratified according to dementia, nutritional status and dependency. Interaction in the dementia and dependency groups was observed. The reasons behind this interaction remain unclear, warranting further studies.
